# Longistatin in tick-saliva targets RAGE

**DOI:** 10.18632/oncotarget.6032

**Published:** 2015-10-08

**Authors:** Anisuzzaman, M. Abdul Alim, Naotoshi Tsuji

**Affiliations:** Department of Parasitology, Faculty of Veterinary Science, Bangladesh Agricultural University, Mymensingh, Bangladesh and Department of Parasitology, Kitasato University School of Medicine, Sagamihara, Kanagawa, Japan

**Keywords:** tick-saliva, receptor for advanced glycation end products (RAGE), longistatin

Ticks are obligatory blood-sucking ectoparasites and while feeding for a single blood-meal, ticks remain attached to hosts by firmly embedding their barbed mouthparts for several days. Like mosquitoes, they cannot canulate an individual blood vessel; rather, they cause extensive damage in vascular beds and underlying tissues leading to the development of a blood pool, a feeding lesion of ticks. In the blood pool, blood and tissue fluid accumulate from where ticks gradually become engorged after several consecutive days of feeding. Extensive tissue damage and development of a blood pool are common features of tick feeding [1, 2]. In fact, a blood pool is a battlefield where a highly organized submicroscopic molecular war takes place among host, vector and invading pathogen, and each axis wants to win to survive. However, ticks can cleverly manage the host responses developed against the biting insult to ensure an adequate blood meal. Tick salivary gland molecules are thought to have pivotal roles in successful feeding on blood meals from hosts [3]. Our group has characterized several potential molecules having critical functions on tick pathobiology, and of them haemangin is worth mentioning as having anti-angiogenic effects on mammalian vasculatures [4].

Recently, we have isolated and identified longistatin, a 15.5-kDa soluble salivary-gland protein with two functional EF-hand Ca^2+^-binding motifs, from the zoonotic tick *Haemaphysalis longicornis* [5]. Longistatin degrades fibrinogen and activates plasminogen, and has been shown to be very closely linked to the blood feeding processes of ticks [1, 6]. In a recent communication [2], we have shown that longistatin bound with the V domain of the receptor for advanced glycation end products (RAGE) in a concentration-dependent manner at a lower nanomolar range (Kd of 72±8 nM) and the binding was ~3 times higher in the presence of Ca^2+^. Longistatin effectively interrupted RAGE-ligand stimulated cellular ROS production in human umbilical vein endothelial cells (HUVEC). Also, longistatin competed with RAGE ligands including various alarmins and effectively prevented RAGE-ligand induced phosphorylation and degradation of IkB-α and inhibited translocation of NF-kB. In addition, we observed that longistatin significantly suppressed *VCAM* 1, *ICAM* 1 and *eSelectin* expression in HUVECs in a concentration-dependent manner. Of the adhesion molecules, the RAGE-ligand caused robust upregulation of VCAM1, which was in turn abruptly attenuated by longistatin.

Furthermore, longistatin significantly decreased the secretion of GCSF and TGF-β into the culture media of HUVECs. We found that glycated-(Gla)-BSA, a RAGE ligand, induced Wt*,* but not *RAGE^−/−^* mouse-peritoneal resident cells (mPRCs) migration while longistatin efficiently abolished the chemotactic responses of Wt mRPCs towards Gla-BSA. Also, longistatin prevented RAGE/ligand axis-driven inflammation in mammals in foot-pad edema and pneumonia models. We detected marked upregulation of *S100B*, *S100A9* and *S100A8* during tick feeding on mammalian skin. However, endogenous longistatin attenuated RAGE-mediated inflammation in hosts during tick feeding. Feeding of *longistatin-*knockdown ticks and control ticks on Wt and *RAGE^−/−^* mice showed massive infiltration of eosinophils and anti-F4/80-specific macrophages at the attachment sites of the knockdown ticks on the Wt*,* but not on the *RAGE^−/−^* mice, confirming that native longistatin, secreted and injected with tick saliva, modulated RAGE-mediated inflammation. Endogenous longistatin down-regulated *IL-5*, *TNF-α*, *Cox2* and *MCP-5* both at mRNA and protein levels.

**Figure 1:**
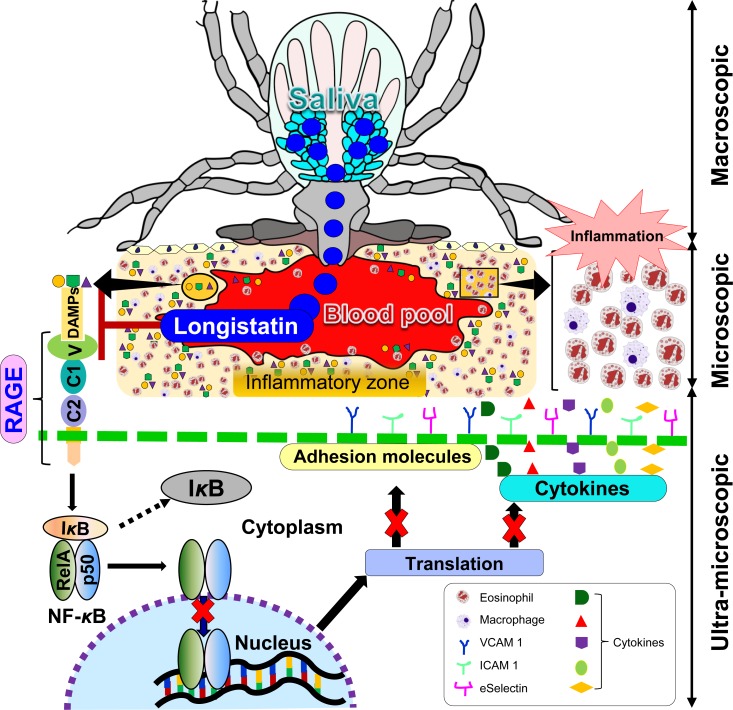
Schematic presentation of roles of longistatin in RAGE-mediated inflammations Ticks cause extensive tissue damage. Dead or devitalized tissues release DAMPs or alarmins, which are responsible for eliciting inflammation, mainly through RAGE engagement, but longistatin prevents interaction of the receptor with the DAMPs. Longistatin effectively suppresses adhesion molecules and relevant cytokine production preventing nuclear translocation of NF-kB.

Since RAGE is constitutively present at a higher level in skin irrespective of age [7] and ticks cause extensive cutaneous damage [1-3], it is quite logical that blockade of the RAGE/ligand axis-driven sustained amplification of inflammatory reactions is beneficial for tick feeding success. Our results clearly suggest that longistatin acts as an antagonist to RAGE and suppresses inflammation during severe tissue injury induced by the biting of ticks and helps in successful feeding on blood. In fact, tick-siaolomics is very interesting to pharmacologists since tick saliva is a potential source of anti-coagulant, fibrinolytic and immunosuppressive agents. These bio-molecules not only have critical roles in the feeding biology of ticks, but also are promising drug targets against thrombotic and immune-mediated disorders in humans [3]. Since our longistatin blocks RAGE, the central player in inflammation, it may be an interesting therapeutic tool against RAGE-regulated diseases e.g.; Alzheimer's disease, psoriasis, diabetic complications and tumorigenesis.
